# Supplementary material for: Does Internet use alleviate household financial vulnerability? An empirical analysis based on panel data from China Family Panel Studies (CFPS)
Source: PLoS One. 2026 Feb 20;21(2):e0343501. doi: 10.1371/journal.pone.0343501 (PMC12923019; doi:10.1371/journal.pone.0343501)
Supplement: Attachment — Submitted filename: review report.docx [file pone.0343501.s001.docx]

**Review of “Does Internet Use Alleviate Household Financial Vulnerability? An Empirical Analysis Based on Panel Data from China Family**

**Panel Studies (CFPS)”**

**(PONE-D-25-20548)**

The authors test the reliability of Tobin's Q, an established proxy for business performance, in emerging economies, focusing on publicly traded non-financial firms in Pakistan from 2015 to 2020. The topic is timely and essential because, while Q is commonly employed in marketing and finance research, its validity in contexts where intangible assets are increasingly important is being questioned.

- The study employs a well-defined panel of 196 enterprises over five years to measure Tobin's Q alongside stock returns and marketing expenditure. Regressions examine the relationship between marketing spending and both Q and stock returns. The research provides a multi-metric evaluation by comparing Tobin's Q to direct performance indicators, such as stock returns. To avoid simultaneity between marketing expenditure and company value, the paper could benefit from addressing endogeneity—for example, by using lagged variables or instrumental approaches.
- This research contributes to the discussion on company performance indicators by experimentally verifying the limitations of Tobin's Q in emerging economies, building on the data of Butta et al. (2021). It supports theoretical critiques of governance ties with Q by Dybvig and Warachka (2015) and Sing (2017). However, the literature evaluation might be expanded by including studies on Q's application in both emerging markets (for example, Yasir Islam's study in Pakistan) and cross-country comparative settings.
- *Ishaq, M., Islam, Y., & Ghouse, G. (2021). Tobin’s Q as an indicator of firm performance: Empirical evidence from manufacturing sector firms of Pakistan. International Journal of Economics and Business Administration, IX, 1, 425-441.*
- The work is well-structured, with clear descriptions of data, methodology, and outcomes. Tables successfully present regression models, but extra robustness checks? such as alternative Q calculations, lagged specifications, or subset analysis by business size? could boost confidence in results. The discussion section might dive more into theoretical mechanics, such as if marketing boosts intangible asset value, hence increasing Q even in the absence of future cash flows. Please break the end and discussion parts into two distinct sections.
- Overall, the study is well-structured, data-rich, and covers an important topic. However, it must make significant improvements to its empirical design, theoretical framing, and external validity before meeting publication standards. Thus, a MAJOR REVISION must be made. Once these issues are resolved, its contribution will be significantly stronger and more persuasive.

Good luck with your paper!
