# Supplementary material for: Does Internet use alleviate household financial vulnerability? An empirical analysis based on panel data from China Family Panel Studies (CFPS)
Source: PLoS One. 2026 Feb 20;21(2):e0343501. doi: 10.1371/journal.pone.0343501 (PMC12923019; doi:10.1371/journal.pone.0343501)
Supplement: Attachment — Submitted filename: Response to Reviewers.docx [file pone.0343501.s002.docx]

**Response to Editors and Reviewers**

Dear Editor and Reviewers,

We sincerely thank the Academic Editor and reviewers for their constructive comments and suggestions, which have greatly helped us improve the quality and clarity of our manuscript. We have carefully revised the manuscript in accordance with the feedback and the journal’s requirements. Below, we provide a detailed, point-by-point response.

**Editor’s Comments**

**Comment 1:** The introduction section should be further enhanced by elaborating on the key gaps and contributions. I would suggest the authors enhance the theoretical argument in the introduction section.

**Response:** We would like to kindly note that in the original version of the introduction, we had already included a preliminary statement regarding the contributions of this study as follows:

- Firstly, utilizing three-wave CFPS panel data to better estimate and identify causal relationships between Internet use and household financial vulnerability, providing novel insights into understanding China’s household financial vulnerability.
- Secondly, expanding the analytical framework by examining mechanisms through income growth, wealth accumulation, and risk management optimization, thereby contributing to a comprehensive understanding of digital technology’s value.
- Thirdly, conducting heterogeneity analyses across regional, urban-rural, and household characteristics to provide empirical evidence supporting precise policy formulation.

In direct response to your suggestions, we have substantially revised the introduction to provide a more explicit and systematic elaboration of the research gaps, theoretical underpinnings, and contributions of our study. Specifically, we have:

- Clearly identified key gaps in the existing literature, particularly the lack of a multi-mechanism framework, insufficient handling of endogeneity, limited heterogeneity analysis, and oversimplified measurement of financial vulnerability.
- Strengthened the theoretical argument by introducing a coherent mediating mechanism framework through which Internet use affects household financial vulnerability—namely, via income growth, wealth accumulation, and risk management optimization.
- Explicitly outlined the marginal contributions of our study in methodological, theoretical, and policy-oriented terms, ensuring they are directly aligned with the identified research gaps.

We believe these revisions have significantly enhanced the clarity, theoretical depth, and overall rigor of the introduction. The revised version now provides a stronger foundation for the empirical analysis that follows.

**Comment 2:** The literature review needs to be enhanced, and up-to-date literature studies should be added to motivate the readers with the background and currency of the subject of the research.

**Response:** We are grateful for this suggestion. In direct response, we have comprehensively enhanced the literature review component within the introduction to better establish the study's background and currency. Specifically, we have:

- Integrated a broader and more up-to-date range of literature: We have incorporated seminal works on the conceptualization of household financial vulnerability (e.g. Loschiavo et al.,2025; Anderloni et al., 2012) and significantly expanded the discussion on digital technology's impact with recent, high-quality empirical studies from China (e.g. Kouladoum, 2025 on human capital; Yuan et al, 2024 on income; Wang et al., 2023 on household financial assets; Zhu et al., 2022 on the digital divide; Yin et al., 2022 on insurance participation). This provides a more robust and contemporary scholarly foundation.
- Strengthened the narrative to motivate the research: The enhanced literature review is now woven into a logical narrative that not only summarizes existing findings but also critically synthesizes them to clearly highlight the evolving research landscape and the specific, timely gaps that our study addresses. This effectively motivates the reader by underscoring the necessity and relevance of our research questions.
- Ensured seamless integration within the PLOS ONE format: Following the journal's style, we have consciously avoided a separate literature review section. Instead, we have meticulously synthesized the reviewed literature throughout the introduction, ensuring it serves to naturally introduce the background, establish currency, and lead into the study's objectives without disrupting the manuscript's flow and conciseness.

We believe these revisions have successfully enriched the background context and scholarly motivation of the study, providing readers with a clearer and more compelling understanding of the research's timeliness and value.

**Comment 3:** The methodology section is good and used different approaches; however, if possible, additional diagnostic tests, including correlations, etc., may further validate the results.

**Response:** We thank the reviewer for this positive feedback and constructive suggestion. We have conducted additional diagnostic tests to further validate our results, as detailed below:

- **Multicollinearity Diagnosis**: We calculated Variance Inflation Factors (VIF) for all independent variables. Results indicate no concerning multicollinearity, with a mean VIF of 1.44 and all individual VIF values well below the common threshold of 10 (the highest being 2.13 for the variable *Region*). This ensures the stability and precision of our coefficient estimates. Detailed results are presented in the last paragraph of the Benchmark regression section.
- **Enhanced Instrumental Variable (IV) Diagnostics**: We have strengthened our IV analysis by: (1) explicitly reporting the first-stage coefficient for our instrument (*Distance → Internet Use*), which shows a statistically significant negative relationship (p < 0.05), empirically validating the relevance condition; (2) reporting the second-stage coefficient for the key variable (*Internet Use → Financial Vulnerability*), which remains significantly negative after controlling for endogeneity; and (3) discussing the results of the Wald test of exogeneity (χ²(1) = 16.64, p = 0.0000), which formally rejects the null hypothesis that Internet use is exogenous, thus justifying our IV strategy.
- **Heteroscedasticity Robustness Check**: To address potential heteroscedasticity in our Tobit model estimates of financial vulnerability degree, we re-estimated the model using bootstrapped standard errors (500 replications). Results confirm that all coefficients maintained their significance, direction, and magnitude compared to original estimates. For example, *Internet use* retained strong statistical significance (p < 0.01) with stable coefficient estimates and similar standard error magnitudes. This confirms that heteroscedasticity does not materially affect our inferences, and we therefore retain the original specification in the main manuscript for consistency and clarity.
- **Proactive Methodological Enhancement**: Beyond addressing reviewer comments, we have added a new subsection (Robustness Checks) to the Statistical Methods section. This addition outlines our comprehensive robustness strategies (including measure replacement and Heckman correction models) and provides readers with a clear roadmap to our robustness validation, thereby enhancing the manuscript's methodological transparency and logical flow.

We believe these additions significantly strengthen the empirical rigor of our study.

**Comment 4:** Please further explain and justify the method used.

**Response:** We appreciate this suggestion. We have thoroughly revised the methodology section to provide a more comprehensive explanation and justification for our choice of econometric models and empirical strategies:

- **Enhanced Justification for Model Selection**: (1) For the Fixed-Effects Panel Logit model: We explicitly state that it was chosen over a Linear Probability Model (LPM) to avoid predicting probabilities outside the [0,1] range and to address heteroscedastic errors. Most importantly, we emphasize its capability to control for unobserved, time-invariant household heterogeneity (e.g. innate risk preferences, cultural attitudes), which is a likely source of omitted variable bias. (2) For the Panel Tobit model: We clarify that it is specifically designed to handle our left-censored dependent variable (financial vulnerability degree censored at 1). We explain that applying OLS to such data would yield inconsistent estimates, and the Tobit model provides a theoretically correct framework for obtaining consistent parameters.
- **Clarification on Variable Selection Approach**: Our control variables were chosen a priori based on economic theory and established literature on household financial vulnerability. We have now added a justification in the control variables subsection, explaining that the selected covariates (e.g. household head's demographics, household characteristics, regional factors) are fundamental determinants empirically proven to influence financial health, ensuring our model is both theoretically grounded and economically interpretable.
- **Deeper Discussion of Endogeneity and IV Choice**: (1) We expanded the discussion on potential endogeneity (reverse causality, omitted time-varying variables) to robustly justify the need for an Instrumental Variable (IV) approach. (2) We provided a more nuanced theoretical justification for our instrument (distance to Hangzhou). We argue that, after controlling for provincial GDP and regional fixed effects, this geographical instrument is plausibly exogenous and likely affects financial vulnerability only through its impact on Internet access quality (relevance condition), thus satisfying the exclusion restriction.

We believe these revisions offer a clearer, more compelling, and accurate rationale for our empirical strategy, demonstrating that our methods were carefully chosen to address the specific statistical challenges and causal identification problems inherent in our research questions.

**Comment 5:** The authors should enhance their discussion section as a separate section. Please provide theoretical and practical policy implications and compare the outcomes to past findings.

**Response:** We are deeply grateful to the editor for this insightful suggestion. In direct response, we have now **added a standalone section titled “Discussion”** to the manuscript. This new section comprehensively addresses the editor's request by:

- **Comparing our outcomes with past findings:** We explicitly discuss how our results confirm prior research on Internet use and income/wealth, while also highlighting our novel contributions—specifically, our multi-mechanism framework and more precise measurement of financial vulnerability that captures both incidence and intensity.
- **Elaborating on theoretical implications:** We interpret our findings through the lens of "digital capital," explaining how Internet use acts as a transformative asset that empowers households and integrates theories from information economics and household finance.
- **Providing detailed practical policy implications:** We moved beyond general statements to propose four specific, actionable policy recommendations focused on pro-equity infrastructure, digital literacy for vulnerable groups, fintech innovation, and differentiated policy interventions.

We believe this new section significantly strengthens the manuscript by deeply contextualizing our findings within the broader scholarly discourse and extracting clear, practical value from our research.

**Comment 6:** The conclusion section needs to be modified. I would recommend adding the key outcomes, followed by limitations and future research directions.

**Response:** We sincerely thank the editor for this crucial recommendation. We have comprehensively revised the original section into a **new** **section Conclusion and Prospects**, which now strictly follows the suggested structure:

- **Key Outcomes**: We begin the section with a succinct and bullet-point-style summary of the three most critical findings from our research, providing readers with a clear take-home message.
- **Limitations**: We openly and honestly discuss the limitations of our study, focusing on data timeliness, measurement challenges, and persistent concerns regarding endogeneity, thereby demonstrating a nuanced understanding of our study's boundaries.
- **Future Research Directions**: We propose several concrete and promising pathways for future scholarship, including investigating types of Internet use, exploring behavioral mechanisms, and studying emerging technologies. This moves the field forward by identifying clear next steps.

We believe these revisions enhance the academic rigor and completeness of our manuscript, providing a balanced and forward-looking conclusion.

**Comment 7:** The organization and formatting of the article require modifications in accordance with standard research design and framework. The authors should keep a uniform formatting for the text and tables/figures throughout the manuscript and follow the guidelines of the journal.

**Response:** We sincerely appreciate this valuable suggestion. In the revised version, we have carefully reorganized sections to align more closely with standard research design and framework, ensuring a clearer logical flow between the introduction, methodology, results, and discussion. In addition, we have applied uniform formatting across all parts of the manuscript, including consistency in headings, references and tables. All elements have been revised to strictly follow the PLOS ONE formatting guidelines, as recommended. We believe these changes have enhanced the overall readability and professionalism of the paper.

**Comment 8:** There are spelling, grammar, and phrasing issues in the manuscript. The authors should revise the language through proofreading.

**Response:** Thank you very much for highlighting this important issue. We have conducted a thorough language revision of the entire manuscript. The spelling errors, grammatical inconsistencies, and awkward expressions have been corrected. To ensure clarity and precision, the manuscript was carefully proofread by multiple team members and cross-checked using professional editing tools. We have also refined phrasing in key sections such as the abstract, methods and results to improve readability and maintain an academic tone throughout. We believe these revisions substantially improve the clarity and overall presentation of the manuscript.

**Reviewer #1**
We would like to note that, as clarified by the Senior Editor, the comments from Reviewer #1 are not related to our submission. In line with the Editor’s instructions, we have not revised the manuscript in response to these comments. We appreciate the clarification and thank the Editor for resolving this matter.

**Reviewer #2**

**Comment 1. Lack of Citations:** A major concern is the absence of citations throughout many paragraphs, which weakens the manuscript’s academic integrity and grounding. The author must ensure that every key claim, concept, or data point is supported by appropriate and recent references. Proper citation not only situates the study within the existing literature but also enhances the reliability and scholarly value of the work. A thorough literature review with accurate citations is fundamental for strengthening the argument and demonstrating awareness of relevant research.

**Response:** We sincerely thank the reviewer for this critical and constructive feedback. We fully agree that comprehensive citations are fundamental to academic integrity and for situating one's work within the existing literature. We have thoroughly reviewed the manuscript and added numerous citations to strengthen the scholarly foundation of our arguments, particularly in the Introduction and Theoretical Analysis sections. Key additions and revisions include:

- **In the Introduction section**, we have integrated a broader and more up-to-date range of literature to substantiate our claims. This includes incorporating seminal works on the conceptualization of household financial vulnerability (e.g., Anderloni et al., 2012; Loschiavo, 2025) and significantly expanding the discussion on digital technology's impact with recent, high-quality empirical studies from China. These new citations cover critical areas such as human capital (Kouladoum, 2025), income effects (Yuan et al., 2024), household financial asset allocation (Wang et al., 2023), the digital divide (Zhu et al., 2022), and insurance participation (Yin et al., 2022), providing a more robust and contemporary scholarly foundation for our study's premise.
- **In the Internet use and household financial vulnerability sectio**n, we have supplemented citations to support key claims regarding the channels through which Internet use affects households. Specifically, we have added references to support the transformative impact of Internet use on the macro-environment and information efficiency (Wang et al., 2024), the role of Internet-based learning in enhancing financial literacy (Zhang, 2025), and the critical function of Internet use in building social capital (Reisdorf et al., 2022).
- **In the Mechanisms of Internet use affecting household financial vulnerability section**, we have strengthened the theoretical groundwork by adding citations to foundational and recent literature at the beginning of each mechanistic discussion. This includes citing recent studies on the general income-elevating effect of Internet use (Ma, 2024) and its role in enabling higher investment returns and wealth accumulation through financial market participation (Ma, 2023).

We believe these revisions, which explicitly cite a body of work from both foundational and cutting-edge (2023-2025) sources, have significantly enhanced the manuscript's academic rigor and demonstrated a deeper engagement with the relevant body of research. We are grateful for this suggestion, which has undoubtedly improved the quality of our work.

**Comment 2. Theoretical Analysis:** It is imperative to explicitly define and discuss the key theories underpinning the study, thereby providing a solid conceptual foundation. Clear articulation of theoretical perspectives will enhance the coherence of the research model and help readers understand the rationale behind variable selection and hypothesized relationships.

**Response:** We are deeply grateful to the reviewer for this insightful and constructive comment regarding the need to strengthen the theoretical foundation of our study. We fully agree that a clearly articulated theoretical framework is crucial for establishing conceptual coherence, justifying variable selection, and solidifying the rationale behind our hypothesized relationships. In direct response to this feedback, we have undertaken a substantial revision of the **Theoretical analysis and research hypotheses section** to explicitly define, integrate, and discuss the key theories that underpin our research. Specifically, we have implemented the following key revisions:

- Incorporated two dedicated paragraphs in the Theoretical analysis and research hypotheses section introducing an integrated framework combining Digital Divide Theory and Household Finance Theory as the core theoretical lenses guiding our analysis.
- Explicitly articulated how other foundational theories, such as Human Capital Theory, Information Economics (transaction costs and asymmetry), and Social Capital Theory, inform our understanding of the specific mechanisms linking Internet use to financial vulnerability.
- Provided a theoretical justification for our control variable selection, explaining that variables related to the household head are grounded in Human Capital theory, while other household and regional factors are motivated by theories of household production and regional economics.

We believe these revisions have successfully provided a solid and explicit conceptual foundation for the study, greatly enhancing the coherence of our research model and the theoretical rationale behind our variable selection and hypothesized relationships. We thank the reviewer for this suggestion, which has significantly improved the theoretical rigor of our manuscript.

**Comment 3. Transition from Theory to Hypotheses:** The shift from theoretical discussion to hypothesis formulation is abrupt and lacks a smooth, logical flow. To improve readability and conceptual clarity, the author should establish clear linkages between theoretical constructs and hypotheses, ensuring each hypothesis is clearly grounded in the preceding theory. This will make the manuscript more structured and persuasive, guiding the reader seamlessly through the research design.

**Response:** We sincerely thank the reviewer for this insightful observation. We agree that creating a seamless transition from theoretical discussion to specific hypotheses is crucial for the manuscript’s logical flow and persuasive power. In response to this comment, we have carefully revised the **Theoretical analysis and research hypotheses section** to ensure each hypothesis is explicitly and logically derived from the theoretical framework and mechanisms discussed beforehand. Specifically, we have implemented the following revisions:

- Added a Transitional Summary Paragraph: We have inserted a new transitional paragraph at the end of the theoretical discussion for each major mechanism. This paragraph briefly synthesizes the theoretical logic and empirical evidence just discussed and explicitly states the purpose of deriving a testable hypothesis from this foundation. This creates a logical bridge between the general theory and the specific prediction.
- Explicitly Grounded Each Hypothesis in Preceding Theory: We have modified the wording of each hypothesis to directly connect it to the theoretical constructs and mechanisms previously elaborated. We now use phrasing that clearly indicates the hypothesis is a logical and testable implication of the theoretical discussion, rather than a standalone statement.
- Improved Conceptual Clarity and Flow: These changes ensure the reader is never left wondering why a particular hypothesis is being proposed. The theoretical rationale is firmly established first, and the hypothesis is then presented as its direct consequence, guiding the reader seamlessly into the research design section that follows.

We believe these revisions have significantly strengthened the manuscript’s structure by providing a clear, logical, and persuasive pathway from theoretical constructs to empirical predictions. We are grateful for this suggestion, which has greatly enhanced the readability and conceptual rigor of our work.

**Comment 4. Justification for Variable Selection:** There is an absence of clear justification for why the main variables were selected for this study. The author should provide a detailed rationale for the inclusion of each variable, grounded in theory and supported by prior empirical evidence. Clarifying the relevance and significance of these variables will strengthen the manuscript's conceptual framework and enhance the study's contribution to the field.

**Response:** We thank the reviewer for this critical observation. We acknowledge that in our original manuscript, the justification for variable selection required strengthening, particularly regarding the theoretical grounding of control variables. While the operationalization of the dependent and independent variables was described, the rationale for their selection—and especially for the comprehensive set of control variables—was not sufficiently articulated with reference to established theory and prior empirical evidence.

In response, we have thoroughly revised the Variable **selection section** to provide a detailed theoretical and empirical rationale for the inclusion of every variable.

- For the dependent variable (Household Financial Vulnerability), we have enhanced the justification for adopting the financial margin and solvency approach—as opposed to simpler debt-based ratios—by grounding it in household finance theory, which emphasizes holistic risk management capabilities and resilience to shocks.
- For the independent variable (Internet Use), we strengthened the rationale for employing a frequency-based composite measure by integrating arguments from digital divide theory, emphasizing that usage intensity—not merely access—is critical for shaping socioeconomic outcomes.
- Most substantially, for each control variable, we now explicitly cite the relevant economic theory—such as human capital theory for household-head attributes, household production theory for demographic structure, and institutional economics for Hukou status—and provide specific references to empirical studies that establish their relevance to household financial vulnerability.

These revisions significantly strengthen the conceptual clarity and theoretical grounding of our empirical model, and we believe they substantially enhance the manuscript's contribution to the literature.

**Comment 5. Validity Testing of Control Variables:** Control variables are incorporated in the analysis but their validity is neither tested nor discussed. It is critical to assess the appropriateness and impact of control variables based on established guidelines such as those proposed by Memo et al. (2024) and Becker et al. (2015). Testing and reporting the validity of control variables will improve the robustness of the results and ensure that the effects of confounding factors are properly accounted for.

**Response:** We sincerely thank the reviewer for raising this crucial methodological point, which we acknowledge was insufficiently addressed in our original manuscript. In direct response to this comment, we have conducted comprehensive validity tests for our control variables.

- First, Spearman correlation analysis confirmed that all control variables are significantly correlated with both the independent variable (Internet use) and the dependent variable (financial vulnerability) in theoretically expected directions. For instance, education level and age showed strong correlations with Internet use, while health status and Hukou type exhibited significant correlations with financial vulnerability. Although regional dummy variables and provincial GDP were highly correlated, the Variance Inflation Factor (VIF) tests (mean VIF = 1.44) confirm that multicollinearity does not bias our estimates.
- Second, we performed coefficient stability tests by estimating models with varying combinations of control variable groups. The results demonstrate that the estimated coefficient for Internet use remains positive, statistically significant at the 1% level, and remarkably stable in magnitude across all alternative specifications. These findings provide robust evidence that our empirical strategy effectively isolates the net effect of Internet use and mitigates concerns regarding omitted variable bias.

To ensure appropriate manuscript length and reading fluency, we have included a summary of these validation results in the main text. The full detailed results are available upon request. We are grateful for this suggestion, which has significantly strengthened the methodological rigor of our study.

**Comment 6. Cross-Sectional Dependence Tests:** The manuscript lacks any mention of cross-sectional dependence tests, which is a significant methodological omission, especially in studies involving panel data. The author should conduct appropriate tests to detect cross-sectional dependence and clearly explain how any identified issues are addressed. This step is vital to ensure the reliability and validity of the empirical results and to avoid biased inferences.

**Response:** We sincerely thank the reviewer for raising this important methodological point. We agree that cross-sectional dependence is a crucial concern in panel data studies, and we appreciate the opportunity to address it more thoroughly.

- In response to the comment, we conducted a rigorous robustness check to assess the potential impact of cross-sectional dependence on our inferences. However, due to the specific characteristics of our dataset and the nature of our preferred nonlinear models (fixed-effects logit and tobit), we encountered two practical challenges in directly implementing the reviewer's suggested approaches: (1) Technical Limitations with Nonlinear Models: Standard tests for cross-sectional dependence (e.g. Pesaran’s CD test) and corrections (e.g. Driscoll-Kraay standard errors) are primarily designed for linear panel models. Their application to nonlinear models like our baseline specifications is not straightforward and lacks established theoretical foundations in standard econometric software. (2) Data Structure: Our dataset is an unbalanced panel with a very large number of households (N > 14,600) and a small number of time periods (T=3). This structure, combined with gaps between survey waves (2016, 2018, 2020), makes implementing tests like the Pesaran CD test computationally prohibitive and Driscoll-Kraay standard errors inapplicable due to the irregular time spacing.
- Our chosen approach to address this issue was as follows: As a robust and conservative alternative, we followed the established practice in applied microeconometrics (e.g. Bertrand et al., 2004) by re-estimating the relationships using Linear Probability Models (LPM) and Linear Fixed-Effects models. This allowed us to compute province-clustered standard errors, which are robust to arbitrary within-province correlation (a common source of cross-sectional dependence) and heteroskedasticity.
- Interpretation of the Results: The results of this robustness check (available from the authors upon request) strongly support the reliability of our main findings. Crucially, the coefficient on our key independent variable, Internet use, remains highly statistically significant (p < 0.01) in both linear specifications. Furthermore, the direction of the effects is entirely consistent with our baseline nonlinear models: (1) Internet use is associated with an increased probability of being financially vulnerable. (2) Yet, it is associated with a decrease in the severity of financial vulnerability. The fact that our core results hold under this alternative estimation strategy, which explicitly accounts for potential cross-sectional dependence, greatly strengthens our confidence in their robustness.
- Regarding the presentation of these results: We have chosen not to include the full tables from these linear models in the manuscript itself to maintain a clear focus on our primary empirical strategy (the nonlinear models) and for the sake of conciseness. Presenting results from a model specification that is not our preferred one could potentially distract the reader. Nevertheless, we have added a detailed description of this robustness check in the manuscript and explicitly state that the results are available upon request. We are, of course, fully prepared to provide the complete output to the reviewer and editor should they wish to examine it.

Thank you again for this insightful comment, which has undoubtedly improved the rigor of our analysis.

**Comment 7. Limitations and Future Research:** A notable gap in the manuscript is the absence of a discussion on the study's limitations and directions for future research. Including a dedicated section that critically acknowledges the study's constraints and proposes areas for further investigation will not only add depth to the research but also guide subsequent studies. This reflection demonstrates scholarly rigor and openness to ongoing inquiry, enhancing the overall contribution of the manuscript.

**Response:** We sincerely thank the reviewer for this constructive suggestion. We fully agree that a thoughtful discussion of a study's limitations and potential future directions is a critical component of scholarly rigor and contributes significantly to the collective research endeavor.

- In direct response to this comment, we have now incorporated a dedicated subsection titled “Limitations” and “Future Research Directions” into the manuscript.
- This new section explicitly acknowledges several key limitations of our study: (1) Temporal Scope: We acknowledge that our data concludes in 2020, preceding the rapid acceleration of technologies like generative AI, and thus may not capture the very latest digital dynamics.(2) Measurement Precision: We critically discuss the potential constraints of our measures for financial vulnerability and certain mediating mechanisms (e.g., social capital), suggesting avenues for refinement.(3) Endogeneity Concerns: While we employed an instrumental variable strategy, we openly discuss the perennial challenge of establishing perfect exogeneity and the potential for unobserved confounding.
- Furthermore, we leverage these limitations to propose several concrete and promising avenues for future research, including:(1) Disaggregating the type of Internet use (e.g. productive vs. consumptive). (2) Unpacking the behavioral “black box” through experimental designs. (3) Exploring next-generation digital technologies (e.g. AI-finance). (4) Conducting cross-country comparative analyses.

We believe this addition adds considerable depth to our manuscript, demonstrates a critical and open scholarly stance, and provides a valuable roadmap for other researchers to build upon this work. We are grateful to the reviewer for prompting this important improvement to our paper.

We believe that the revised manuscript has addressed all the concerns raised and now fully meets the requirements of PLOS ONE. We are sincerely grateful to you and the reviewers for the time and constructive feedback, which have substantially improved the quality and clarity of our work. We look forward to your favorable consideration of our submission.

Sincerely,

Heyu Li

E-mail: [heyu.li@foxmail.com](mailto:heyu.li@foxmail.com)

On behalf of all co-authors
