# Supplementary material for: Does Internet use alleviate household financial vulnerability? An empirical analysis based on panel data from China Family Panel Studies (CFPS)
Source: PLoS One. 2026 Feb 20;21(2):e0343501. doi: 10.1371/journal.pone.0343501 (PMC12923019; doi:10.1371/journal.pone.0343501)
Supplement: Attachment — Submitted filename: Response_to_Reviewers_auresp_2.docx [file pone.0343501.s003.docx]

**Response to Editors and Reviewers**

Dear Editor and Reviewers,

Thank you for your careful assessment of our revised manuscript entitled “Does Internet use alleviate household financial vulnerability? An empirical analysis based on panel data from China Family Panel Studies (CFPS)” (Manuscript ID: PONE-D-25-20548). We appreciate your constructive guidance and are pleased to submit a further revised version that addresses the remaining minor issues prior to acceptance.

In this revision, we have implemented the following updates: (1) added an introductory sentence at the start of the abstract; (2) reinstated and provided the keywords immediately after the abstract; (3) added a figure illustrating the mechanism analysis; (4) restored numbering for all headings and subheadings; and (5) proofread the manuscript to correct minor grammatical and phrasing issues.

We respond to each point below:

**Editor Comment 1) The authors should add an introductory sentence or two at the start of the abstract.**

**Response:** We have revised the abstract by adding an introductory sentence at the beginning to better motivate the research question and contextualize the study.

**Change made:** An opening sentence has been inserted at the start of the Abstract section.

**Editor Comment 2) Please add the keywords after the abstract. The authors have removed the keywords in the revised version.**

**Response:** We have added the keywords immediately after the abstract, as requested.

**Change made:** We inserted the keywords in the manuscript right after the abstract. The keywords are:

Keywords: Internet use; Household financial vulnerability; Income growth; Wealth accumulation; Risk management capability.

**Editor Comment 3) If possible, please add a figure highlighting the research design/framework.**

**Response:** We agree that a visual summary improves clarity. Accordingly, we have added a new figure that highlights the overall research design and analytical framework.

**Change made:** We added Figure 1, titled “Flowchart of Mechanism Analysis”, along with an explanatory caption in the manuscript.

**Editor Comment 4) The authors have removed the numberings. Please add numberings to the headings and subheadings.**

**Response:** We have reinstated numberings for all headings and subheadings to improve readability and navigation.

**Change made:** Section and subsection numberings (e.g., 1, 1.1, 1.2, …) has been restored throughout the manuscript.

**Editor Comment 5) There are minor grammatical and phrasing issues. The authors should revise the language through proofreading.**

**Response:** We have carefully proofread the entire manuscript and corrected minor grammatical errors, awkward phrasing, and inconsistencies in style and terminology.

**Change made:** Language edits have been implemented across the manuscript to improve clarity and academic tone while preserving the original meaning.

**Reviewer Comments**

We also note that Reviewer #1 indicated satisfaction with the revisions and recommended acceptance without raising any further specific comments requiring a point-by-point response. Reviewer #2 provided no additional response in this round; therefore, there were no reviewer-specific comments for us to address beyond the editorial requests above.

Thank you again for your time and consideration. We hope that these revisions fully address the remaining issues, and we would be grateful for your confirmation that the manuscript is now suitable for acceptance.

Sincerely,

Heyu Li (on behalf of all authors)

[heyu.li@foxmail.com](mailto:heyu.li@foxmail.com)
